# Telehealth for the cognitively impaired older adult and their caregivers: lessons from a coordinated approach

**DOI:** 10.2217/nmt-2020-0041

**Published:** 2020-11-10

**Authors:** Erica F Weiss, Rubina Malik, Teresa Santos, Mirnova Ceide, Jason Cohen, Joe Verghese, Jessica L Zwerling

**Affiliations:** ^1^Department of Neurology, Montefiore Medical Center and Albert Einstein College of Medicine, Bronx, NY 10467, USA; ^2^Department of Medicine (Geriatrics), Montefiore Medical Center and Albert Einstein College of Medicine, Bronx, NY 10467, USA; ^3^Department of Psychiatry, Montefiore Medical Center and Albert Einstein College of Medicine, Bronx, USA

**Keywords:** advance directives, caregiver stress, coordinated care, dementia, models of care, telemedicine

## Abstract

The Covid-19 pandemic forced providers to alter their delivery of care to special populations, including older adults with cognitive impairment. The Montefiore–Einstein Center for the Aging Brain, a specialty multidisciplinary center for the evaluation and management of patients with neurodegenerative disorders, developed a coordinated approach (Coordinated Care At Risk/Remote Elderly program [CCARRE]) to reach our diverse population during the initial Covid-19 crisis in New York City, USA. In the tele-evaluation of the first 85 patients seen with CCARRE, we recognized unique factors that could improve patient care, lessen burden and optimize access to community resources. Lessons learned from the experience are shared.

**Practice points**

- Video visits allow the provider to ‘see’ their patient without increasing infection risk during the Covid-19 pandemic. Starting the visit on the phone and transitioning to video after rapport is established improved transition/comfort with video use.
- Video visits can be used to complete a real-time safety evaluation and address safety needs.
- Concerns about Covid-19 offer an opportunity to address advance care planning and goals of care.
- Caregiver stress and caregiver needs are equally important, particularly as living situations and caregiver demands change in response to the public health crisis.
- Alternatives to social, physical and cognitive stimulation in daily activities and consideration of virtual events/activities as individuals were primarily staying home are welcomed by patients and caregivers.
- Social determinants of heath need to be addressed before patients and caregivers can focus on cognitive concerns.
- Cultural, social and language factors should be considered not only as part of component evaluations and care of neurodegenerative diseases but also are important in the use of telehealth.
- Multidisciplinary approaches improve care and connection to community-based organizations can be used to support multidisciplinary and independent providers.

The unprecedented scale and nature of the Covid-19 pandemic has forced dramatic changes in medical care delivery. Safety concerns regarding viral transmission have led to restrictions in face-to-face clinical encounters and increased use of telemedicine approaches. As providers and patients navigated the transition to telemedicine over the past few months, certain populations, particularly older adults from health disparate populations, were, and to some extent continue to be, at increased risk of being left behind [1,2].

Under the best of circumstances, the care of the older adults, especially those with cognitive impairment, is a challenging task, complicated by barriers to care, limited resources, medical complexities and caregiver factors [3]. Language barriers, sensory difficulties, poor health literacy and poorly controlled chronic medical conditions make telemedicine challenging. Core geriatric issues, such as weight loss, malnutrition, polypharmacy, sensory deficits, gait and balance problems, sleep dysfunction, chronic illnesses, safety, home care needs, behavioral symptoms, as well as caregiver stress, are prevalent in patients with cognitive impairment and are potentially exacerbated by the Covid-19 pandemic. Cultural factors and emotional functioning further interplay with the fear and isolation being experienced by all, and become an increasing area of difficulty for socially isolated, culturally diverse and health disparate populations.

The Covid-19 pandemic served as an opportunity to evaluate current healthcare delivery systems and to create adaptations to the new situation. To effectively address the needs of our cognitively impaired older adults as well as their caregivers, we adapted our model and provided collaborative telemedical care to new and established patients with cognitive issues. We describe the ‘Coordinated Care At Risk/Remote Elderly’ program (CCARRE) to demonstrate a unique approach to reaching our culturally diverse and vulnerable population using telehealth, and share some of the lessons we learned as a result of early difficulties we faced.

## Dementia model

The Montefiore–Einstein Center for the Aging Brain (CAB) is a specialty referral center for the evaluation and management of cognitive complaints in older adults. The CAB assessment protocols have been previously described [4]. In brief, all patients referred to the CAB have clinical evaluations performed individually by a geriatrician, a neuropsychologist and a neurologist. Social work, physiatry and geriatric psychiatry referrals are available as needed. Our behavioral care manager reviews each case. However, during the Covid-19 pandemic in-person office visits were suspended and providers faced redeployment. To meet our patients’ needs and to reduce patient and caregiver burden, the multistep process was condensed into a CCARRE visit conducted via telephone or HIPPA-compliant secure video platforms.

## CCARRE televisit model

The CCARRE team included a bilingual/bicultural social worker and a neurologist conducting the telehealth visit with a patient and the identified healthcare proxy or caregiver on record.

New and established patients were seen as part of CCARRE. All patient/caregiver dyads were offered secure video visits, but patient/caregiver preference and their technological limitations often necessitated that visits were conducted using telephone or live video technology. After introductions, confirmation and consenting of patient as required by local statutes, the visit included a comprehensive review of medical, social and psychiatric history with verification from the caregiver. Consistent with our established CAB model [4], the CCARRE medical review focused on falls/mobility issues, weight loss, sensory impairment, sleep/ behavioral disturbances and medication review (focused on limiting polypharmacy and addressing inappropriate medication use). Social determinants of health, including food and housing insecurity were addressed, and the patient-caregiver dyad was assessed for domestic violence and elder neglect/abuse. Caregiver stress was evaluated, and advance care planning, emergency planning and respite needs were discussed.

The neurologist’s focused examination included review of instrumental and activities of daily living, and review of neuropsychiatric symptoms. The neurobehavioral exam evaluating orientation, concentration, memory, language, praxis, mood, affect, insight and judgment as well as thought content/process was completed. Validated cognitive and depression/anxiety assessment tools were engaged at each visit. Telephone visits used the Blessed Information Memory Concentration Test (BIMC [5]), and/or the telephone version of the Memory Impairment Screen (MIS-T [6]). All video visits employed the Picture Memory Impairment Screen (PMIS [7]). The BIMC and the PMIS are the screening tools used by the CAB as part of routine cognitive assessment before the pandemic, with the PMIS providing a culture-fair and literacy-independent memory screen in our ethnically diverse population [8].

When video was available, a limited cranial nerve, motor exam and coordination/balance and gait exam were conducted. Modifications to the in-office exam included asking the caregiver to place the video device on the floor to assess gait, which allowed for examination of overall tone, coordination and viewing of adventitious movements or neglect syndromes.

At the end of the televisit, the neurologist prepared a comprehensive plan of care that addressed the patient’s cognitive status and any medical, behavioral or psychosocial issues. The CCARRE plan was discussed with the patient and caregiver, and reports were sent to the referring physician and the primary care provider (if not the same as the referring physician) to optimize management of co-morbidities. Feedback from patient, caregiver and primary care providers were informally solicited. Referrals to community-based organizations and clinical trials were made, as appropriate.

Approaching the patient/caregiver dyad through this multidisciplinary collaboration facilitated the delivery of comprehensive caregiver/patient education and comprehensive, coordinated treatment plans, but also revealed unmet needs and concerns elicited by Covid-19.

## The CCARRE outcomes

In the 2 months that New York City and our health system were in the peak of the Covid-19 pandemic (18 March–18 May 2020), 85 patients were evaluated as part of CCARRE. The demographic makeup of the patients is provided in Table 1. Of the patients seen, 27 (32%) were new patients to our center – although some (11) had completed the geriatric or neuropsychology component of the visit prior to the pandemic or prior to the CCARRE visit. Patient were referred to the CAB from the community, primary care physicians or community neurologists as described in our CAB protocol paper [4]. Most (>90%) of the patients seen were scheduled prior to pandemic and their visits were converted to televisits by front desk staff. Of the 103 visits scheduled during this time period, we had a 17% no-show rate (n = 18). Patients who ‘no-showed’ for visits were more likely to be established (88%), female (78%), African–American/black (44%) and high school graduates (38%). Of the 18 no-show visits, five individuals were eventually seen within the 2 months. Reasons for no-show were not formally documented but informally included: forgetting the appointment, patient illness/hospitalization and patient death. In addition to referral questions and questions about the ‘virus’, common concerns of patients and caregivers included: running out of medications; when to escalate care to the hospital if someone became ill; and if living together as an intergenerational family posed additional risk.

**Table 1.** Demographics of patients seen for Coordinated Care At Risk/Remote Elderly program.

	n = 85
Age (mean, SD)	76.33 (9.51)
Gender (n, % female)	47 (55.3%)
Ethnicity (n, %)	
Hispanic/Latino/Latina	50 (58.8%)
African–American/Black	16 (18.8%)
White/Caucasian	15 (17.6%)
Asian	3 (3.5%)
Primary language (n, %)	
English	53 (62.4%)
Spanish	30 (35.3%)
Other	2 (2.4%)
Education completed (n, %)	
Less than 8 years	24 (28.6%)
8–11 years	22 (26.2%)
High school graduate	12 (14.3%)
Some college	9 (10.7%)
College graduate	9 (10.7%)
More than college	8 (9.5%)
Insurance/payor information (n, %)	
Medicare only	44 (52.4%)
Medicaid only	7 (8.3%)
Medicaid and medicare	26 (31.0%)
Private insurance	7 (8.3%)

SD: Standard deviation.

The CCARRE visits took an average of 50 min for new patients and 35 min for established patients. These visits resulted in 89 referrals to community-based service organizations (such as the Alzheimer’s Association, Caring Kind etc.) as well as referrals to internal services within our health system, including telehealth counseling, physical therapy and physiatry.

Patient, caregiver and physician postevaluation feedback, collected informally, was positive. Referring providers, many of whom were deployed or out office due to Covid-19, found the CCARRE reports extremely helpful and replied to evaluation reports with appreciation and a direct follow-up plan for patients/caregivers. Caregivers reported that the televisits adequately addressed all concerns that they had at the time of the visit; most reported wanting to return to in-person visits once safe but noted comfort with having their follow-up visits remaining virtual until that time.

As the CAB team discussed our experiences and early tribulations in the implementation of CCARRE visits, we recognized factors and techniques that improved our ability to provide care and address patient/caregiver needs. These ‘lessons’ are offered to assist others faced with transitioning to telehealth.

## Lessons learned

### The televisit approach

Although video visits provide clinical data and an improved ability to build rapport that telephone encounters alone cannot, there are challenges to the completion of televisits with older adults with cognitive impairments and their caregivers. Generally, older adults are less comfortable with technology, may lack access to the necessary technology and may have sensory difficulties (e.g., reduced hearing or vision) that impede in the use of technological devices [1].

### Start on the phone & move to video

Even when patients had access to video capabilities (smart phones or tablets), many initially refused video visits offered by the support staff calling to confirm their appointments. We found that starting the visit on the phone, establishing rapport and then asking the patient and caregiver to switch to video increased comfort and compliance with video visits in most of the patients/caregivers. Patients and caregivers who had participated in a previous video visit were more likely to agree to a video visit.

### Use the video visit to conduct a ‘real-time’ safety evaluation

Video telehealth visits allow a look into the patient home that office visits rarely provide. We used these ‘peeks’ to address physical and other safety concerns in our high-risk population. For example, the CCARRE gait evaluations revealed navigational obstacles in the home that increased risk of falls and injury. Medication review allowed the provider to see how medications are stored and delivered. Invitation into the home environment allowed for more directed conversations about social determinants of health (see below) and an ability to evaluate possible elder neglect. The video visit allowed the CCARRE team to flag patients who needed home assessments and prioritize those who needed urgent follow-up as access to home evaluations was limited.

### The ‘virus’ approach

Understandably, a frequent topic raised by patient-caregiver dyads was fear of the ‘virus.’ This ranged from fear of getting sick, to being isolated at home, to anxiety about possible hospitalizations related to Covid-19 infection or other medical issues. These practical concerns allowed the CCARRE team to address important topics that we highlight here.

### Address advance care planning & goals of care

Advance care planning is an important component of managing cognitive impairment, however, it is a topic that many patients and families struggle with. The Covid-19 pandemic made this conversation even more crucial. As hospitals limited visitors, caregivers voiced concern of impaired loved ones being alone in the hospital. Also, reports of higher risk of infection in older adults made caregivers and patients with mild cognitive impairment consider what would happen if they were to become ill. These fears allowed the CCARRE team to elucidate and facilitate goals-of-care discussions, assist with advance care planning documentation and help families guide their own care. Our goals-of-care discussions with the patients and caregivers empowered them and helped our colleagues on the front lines.

### Address caregiver stress & caregiver needs, including providing behavioral management techniques

As the pandemic caused individuals to ‘pause’ (‘shelter in place’) in the NY State area, patients and caregivers were now spending increased time with family members in enclosed (and often small) environments. As stress levels increased for all, our discussions with caregivers revealed two factors that directly increased caregiver stress and an additional factor that lessened stress for some.

Increased concern about virus transmission forced many direct caregivers to isolate from their support systems and refrain from their self-care routines (exercise, going to their own physicians, socializing etc.) leading to increased stress and difficulty coping. While normalizing and supporting the caregivers’ feelings, we worked to connect them to virtual support groups, including our own center’s support group and individual therapy services. We also emphasized the development of alternative self-care tactics and reviewed stress management techniques during the visit.
Primary caregivers who were previously typically outside the home, voiced concern and stress as they spent more time with their loved one and recognized neuropsychiatric manifestations of dementia. This offered us a unique opportunity to provide and reinforce education about common behavioral manifestations and teach techniques for how to manage those manifestations (see below).
+ An unexpected benefit that some of our caregivers and patients reported included a decline in behavioral outbursts and a decrease in depression and anxiety in several patients. For more impaired individuals, as their routine care was now performed consistently by one family member, there was less agitation and aggression. In individuals with mild cognitive impairment, especially those with concurrent mood symptoms who previously lived alone, the pandemic led to intragenerational households and some patients reported improved mood symptoms and decreased anxiety as they were now residing with a family member.

### Address daily activities – cognitively, socially & physically appropriate things patients can/should be doing

There is wealth of literature supporting cognitive, social and physical activity as techniques to slow cognitive decline [9,10]. As patients and caregivers became increasingly homebound, many struggled to adapt their routines. Caregivers and patients benefited from information about available virtual community resources (some of which were culturally specific – a factor that tends to increase adoption), and discussion with their referring providers about what they could or could not do safely. We also used this time to highlight nonpharmacological treatment options/techniques to address behavioral manifestations. These included patient-centered leisure activities, psychological support therapy, distraction techniques for caregivers and music/art therapy.

### The multidisciplinary approach

We have been supporters of a collaborative multidisciplinary approach to clinical care of older adults with cognitive impairment for a long time, and our collective work at the CAB has reinforced the benefit of having multidisciplinary specialties/ subspecialties work as a team to address patient and caregiver diagnoses, management and needs. By including a bilingual/bicultural social worker who is also a certified medical interpreter on our CCARRE visits, we were able to capture and address unique needs that were at times more urgent than some of the cognitive concerns for which the patient was being seen. While we understand that not all providers have access to embedded social work services, we offer the following key aspects that became evident.

### Address social determinants of health

Food insecurity and fear of loss of housing were concerns raised by several caregiver–patient dyads as senior centers stopped serving lunch and caregivers/providers lost jobs due to the pandemic. While we could not address the underlying societal factors, we were able to review access to healthcare, access to food, crime/violence (including gun possession), environmental conditions, health literacy and social connectiveness with each dyad and make appropriate links to address acute needs. The CCARRE team was able to connect families to local free food sources to ensure that the patient and caregivers could have appropriate nutrition.

### Consider cultural/racial factors & language barriers

Cultural, ethnic and racial factors impact patient engagement in care and the trust of medical providers [11]. Culturally competent care of the older adult is an important component of our work at the CAB and should be a component of all neurodegenerative evaluations/diagnoses. Our catchment area is urban and multicultural, and patient–caregiver dyads speak a variety of languages. A recent report indicated that our health disparity population and catchment area in the Bronx and Westchester counties had the highest rates of Covid-19-related hospitalizations and death in New York [12]. We approach patients in their self-identified language and use culturally sensitive norms and knowledge to ascertain change from optimal abilities. As we transitioned to telehealth, we learned that many of our patients who emigrated to the USA were comfortable with non-HIPAA compliant video communication services (particularly ‘WhatsApp’), which they used to communicate with family ‘back home’. Patients/caregivers were more likely to agree to video visit when given the option to join via their preferred service (when it was allowed with the state of emergency HIPAA waiver), and some of our colleagues began to describe our internal video communication software as similar to ‘WhatsApp’, which reduced barriers to join. We would be amiss to overlook the impact of reports of increased susceptibility and incidence of Covid-19 in non-Caucasian individuals and the concurrent increased recognition of ‘systemic racism’ that occurred during this time period. Although beyond the scope of what most people think about when they imagine a neurological/neurodegenerative evaluation, both are factors that are impacting our patients.

### Link with local community-based organizations to establish seamless connections

Our established relationship with local community-based organizations (area chapters of the Alzheimer’s Association, Caring Kind etc.) allowed the CCARRE team to easily make referrals for the concrete needs of the patient and caregiver.

Organizations like these provide caregiver support services (even virtually) and can address patient/caregiver questions that are beyond the scope of medical practice. For providers who do not have embedded social workers, these organizations offer resources to answer caregivers’ and family members’ questions and concerns about benefit eligibility, pooled trusts, managed long-term care, nursing home placement and the availability of home care services.

## Conclusion

As Covid-19 impacted healthcare systems, patients and providers, and as healthcare was taken out of the office and into the telehealth space, older adults, particularly those with cognitive impairment, were at a risk for getting left behind. Our coordinated team transition to telehealth, while initially challenging, led to new insights into areas of urgent need for our diverse, cognitively impaired population and opened avenues to address factors that impact both our cognitively impaired patients and their caregivers. As cases of Covid-19 continue to rise across the USA, we support the use of telehealth as a complementary tool to in office assessments for our most vulnerable populations. The CCARRE model provided access to the healthcare system during a time when it was challenging to make contact with the primary care physicians who were deployed. Lessons learned from this crisis demonstrate the use of technology platforms and incorporation into routine practice and the benefits to caregivers/patients.

## Future perspective

Televisits and the use of video technology in healthcare will outlast the current public health emergency. Telehealth has the potential to provide access to care/services to patients who otherwise would never receive it, especially those who live in resource-limited locations, those far from their nearest providers and those who are homebound. Additional research will be necessary to validate standard measures/tools and best practices.
